# Environmental factors: a “missing link” in COVID-19

**DOI:** 10.1007/s40629-021-00170-w

**Published:** 2021-04-09

**Authors:** Stefanie Gilles, Athanasios Damialis, Claudia Traidl-Hoffmann

**Affiliations:** 1 Department of Environmental Medicine, Faculty of Medicine, University of Augsburg, Neusaesser Str. 47, 86156 Augsburg, Germany; 2 CK-CARE, Christine Kühne Center for Allergy Research and Education, Davos, Switzerland

**Keywords:** COVID-19, Pollen, Environmental factors, Temperature, Humidity

The main driving factor in viral infections, especially in the absence of herd immunity as in severe acute respiratory syndrome coronavirus 2 (SARS-CoV-2), is the respiratory uptake of virus-containing particles that are produced when breathing, coughing, speaking, singing and sneezing. Whether an infection then occurs depends particularly on personal risks and the concentration of infectious viruses. Mobility, “super spreader” events and the recent appearance of highly infectious virus variants have contributed significantly to the increased incidences of corona virus disease 2019 (COVID-19).

Apart from pathogen-derived, individual risk factors and sociodemographic influences, environmental factors such as climate, weather, air quality should be given greater consideration towards the spreading of viruses.

**Fig. 1:**
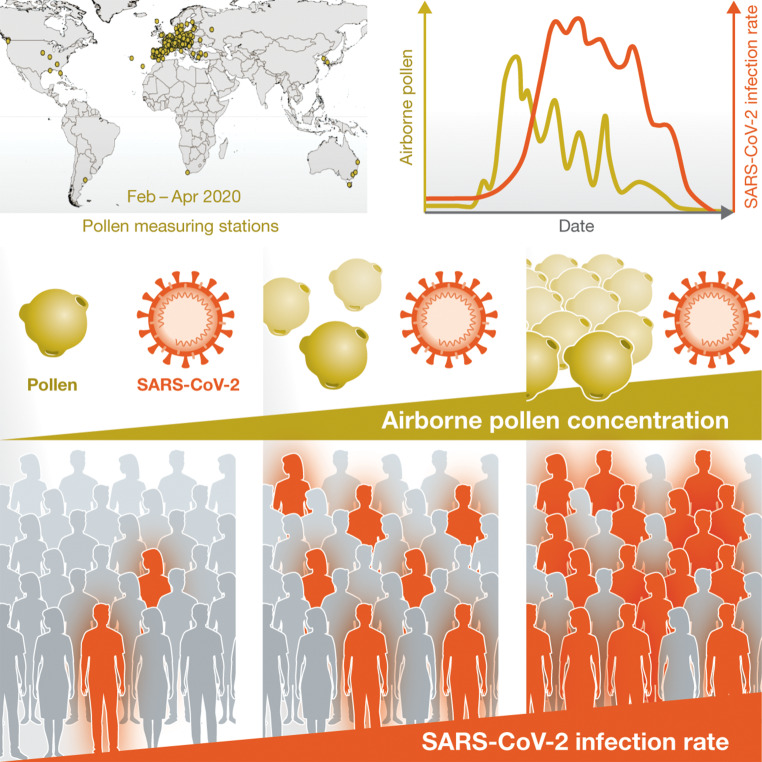
Schematic representation of the study design and key findings of the study by Damialis et al. [1]. Data on pollen concentrations, weather, severe acute respiratory syndrome coronavirus 2 (SARS-CoV-2) infections, population density and lockdown measures were collected from 1 January to 8 April, 2020. The data were from 248 monitoring sites across 31 countries on 5 continents. Apart from the expected protective effect of lockdowns, significant and positive correlations of SARS-CoV‑2 infection rates were observed with environmental factors (higher pollen concentrations, warmer and drier weather) during the study period.

In a most recently published data-oriented study, a link was made between SARS-CoV‑2 infection rates and airborne pollen concentrations [1]. The study data consisted of pollen concentrations and SARS-CoV‑2 infection numbers across 31 countries from both hemispheres and on 5 continents. The key finding was a significant and positive correlation between SARS-CoV‑2 infection rates and environmental factors in the spring of 2020 (study period: 1 January to 8 April 2020; Fig. 1). Besides sociodemographic effects (population density, lockdown), airborne pollen concentrations, air temperature and relative humidity were identified as important modulators of the infection rates within the study period. Pollen concentrations were correlated positively with infection rates in the majority of the examined countries, in synergy also with temperature and humidity. Notably, at sites with low pollen concentrations during the study period, e.g., in the Southern hemisphere or in Northern Europe, most frequently in colder, continental climates, no correlation with pollen was found, and the infection rates were overall still lower than at sites with high pollen concentrations.

**Fig. 2:**
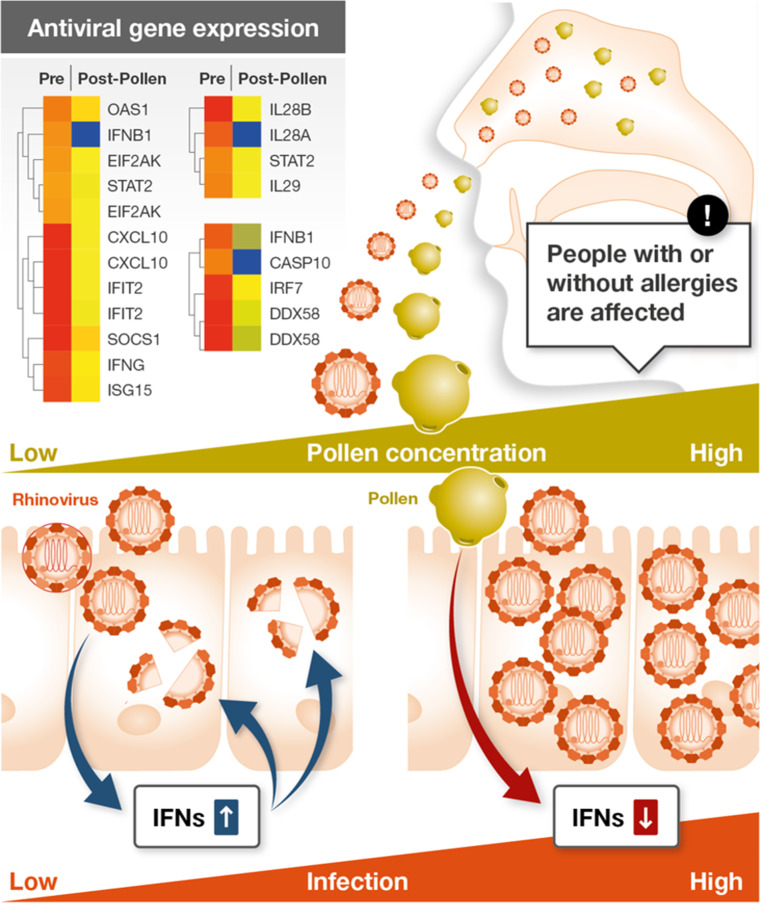
Pollen diminish the antiviral defense of the airways, as published in the study by Gilles et al. [2]. Nasal instillation of pollen leads to down-regulation of genes of the innate antiviral immune response in the nasal mucosa. This was shown in pollen allergic and nonallergic individuals. In human nasal and bronchial epithelial cell cultures derived from donors with and without a pollen allergy, co-exposure to rhinovirus and birch or grass pollen led to diminished antiviral type I and type III interferon responses and increased viral replication. *IFNs* Interferons

The study on COVID-19 and pollen was initiated at the time when SARS-CoV‑2 infection rates began to rise exponentially in the bulk of the Northern hemisphere, coinciding with a major warm and dry weather spell with high pollen concentrations in mid-March 2020. The concept of testing for a potential relationship of airborne pollen with SARS-CoV‑2 infections was based on recent findings published only a few months before, in November 2019 [2]. There, we showed that pollen compromise the innate antiviral defense of airway epithelia by diminishing antiviral type I and type III interferons (Fig. 2). This was concluded from in vitro and in vivo experiments on the co-exposure to human rhinovirus and respiratory syncytial virus and different pollen types. Among other findings, this study found a positive and significant relationship between springtime rhinovirus infections and airborne birch pollen concentrations in a large Swedish cohort (>20,000 patients with respiratory infections from 2011–2013).

When patients with allergic asthma are exposed to “their” triggering allergy-inducer, they have lower levels of antiviral interferons, which is thought to account for an increased susceptibility to viral infections and exacerbations [3, 4]. However, according to current epidemiological data, there is no evidence that individuals with atopic asthma are at increased risk for SARS-CoV‑2 infections or severe COVID-19. For allergic rhinitis patients, the findings appear to point towards the same direction; however, no study has stratified the risks of allergic and nonallergic individuals by the pollen season yet.

In contrast to the above-mentioned immunosuppressive effect of pollen—via a diminished antiviral interferon response—lies the finding that the expression of the main SARS-CoV‑2 entry receptor, angiotensin-converting enzyme 2 (ACE2), is down-regulated in nasal epithelium of atopic asthma patients. This could lead to decreased uptake of SARS-CoV‑2 and lower susceptibility for a severe disease course [5, 6]. Studying in more detail the expression kinetics of ACE‑2, viral coreceptors and proinflammatory as well as antiviral cytokines during the course of SARS-CoV‑2 infections will help to resolve conflicting findings.

The recent publications highlight that environmental factors have to also be added to the equation for COVID-19. The challenge for a future quantification of environmental effects on COVID-19 lies in setting up dedicated, large-scale observational studies. This means the close biomonitoring of clinically (and genetically) well-characterized patients to assess their comprehensive “exposome” in a wide spectrum of environmental regimes. Moreover, the seasonality of environmental factors has to be taken into account when attempting to isolate potential signals in the statistical noise, so as to be able to assess effect sizes in the correct time window per season.
